# Age is not a significant predictor of survival in patients with IDH-wildtype glioblastoma that undergo gross total resection and complete adjuvant chemoradiation

**DOI:** 10.3389/fonc.2025.1657867

**Published:** 2025-09-10

**Authors:** Jeffrey W. Cozzens, Noah B. Drewes, Kristin R. Delfino, Kayla L. Chin, Devin V. Amin, Barbara C. Lokaitis, José A. Espinosa, Breck A. Jones, Leslie J. Acakpo-Satchivi, Hayan Dayoub, M. Bruce Frankel, Edem Agamah, Krishna Rao, C. Matthew Bradbury, John Gao

**Affiliations:** ^1^ Division of Neurosurgery, Southern Illinois University School of Medicine, Springfield, IL, United States; ^2^ Center for Clinical Research, Southern Illinois University School of Medicine, Springfield, IL, United States; ^3^ Springfield Clinic, Springfield, IL, United States; ^4^ Simmons Cancer Institute, Southern Illinois University School of Medicine, Springfield, IL, United States; ^5^ Department of Pathology, Springfield Memorial Hospital, Springfield, IL, United States

**Keywords:** glioblastoma, age, prognosis, frailty, survival

## Abstract

**Background:**

Older age is often cited as a negative prognostic factor for individuals with glioblastoma, but it is unclear if this is true when other prognostic factors are equalized.

**Methods:**

This study is an observational, single-center retrospective analysis of data from consecutive individuals with histologically identified high-grade glioma prospectively accumulated for a registry of all neurosurgical operations in our region from 2010 to 2024 (15 years). Data concerning histology, survival, IDH mutations, MGMT methylation status, extent of resection, frailty (measured by m-Fi-5 index) and subsequent adjuvant treatment (radiation and chemotherapy) were all recorded. Statistical analysis was performed on selected groups with Kaplan-Meier survival analysis, Student’s t-test and multivariable Cox proportional hazards regression.

**Results:**

There were 270 individuals who underwent a neurosurgical procedure resulting in a histopathological diagnosis of glioblastoma. The data from a select group of 91 individuals were examined where all individuals had tumors with IDH-wildtype, gross total resection, and treated with chemoradiation. When univariately assessing for the impact of age on survival, no significant association was found (p=0.5380). After adjusting for MGMT methylation status and frailty, age remained insignificantly associated with overall survival (p=0.4009).

**Conclusions:**

Age does not seem to be a factor in overall survival for glioblastoma when all the other prognostic factors are equalized. The idea that younger age is a positive prognostic factor is probably the result of more frequent IDH-mutant tumors in younger patients, increased incidence of frailty in older patients and the unwillingness of healthcare providers and patients/families to aggressively treat older patients.

## 1 Introduction

Among the various prognostic factors associated with longer survival in patients with glioblastoma (GBM), the influence of age at diagnosis has been most frequently cited (1–11). The common finding is that younger age correlates with longer survival, but there is rarely any explanation for this phenomenon. It is unclear how an individual’s chronological age by itself could be related to the growth or the biological response to treatment of a glioblastoma. Yet the association of better prognosis with younger age is well-accepted (12, 13).

In the past few decades there has been an increasing interest in the health of older individuals as their population has increased (14, 15). Glioblastoma is a tumor that tends to arise in older individuals with a mean age of onset at 64–67 years-old and therefore the issue of cancer treatment in an older population is particularly relevant in this group (11, 16–20).

There are several other factors affecting cancer prognosis and treatment such as medical or socio-economic factors. For example, the impact of medical comorbidities can accumulate and lead to a less favorable treatment outcome. Older individuals who do not suffer from these comorbidities may better withstand the effects of aggressive treatment of their tumor (21, 22). Similarly, older individuals considered to be at the “end of life” may elect to not receive aggressive cancer treatment or may even be denied treatment by their family or care givers (23–25). It is unclear whether these factors are responsible for the observed poorer prognosis in older individuals with glioblastoma.

This study is intended to examine whether older age, independent of all other factors, is truly a negative prognostic factor for individuals with glioblastoma. To do this, our goal was to examine the independent effect of age in a clinically relevant, well-defined population receiving similar standard care.

## 2 Methods

### 2.1 Study design

This study is a cross-sectional, observational, single-center retrospective analysis of data from consecutive individuals with histologically identified high-grade glioma prospectively accumulated for a registry of all neurosurgical operations in our region from January 2010 to December 2024 (15 years).

This study was reviewed and approved by the local Institutional Review Board and Ethics Committee (IRB)(approval number 18-178). The design of the study, the data gathering and analysis and the reporting of the study all conformed to the Declaration of Helsinki of 1975. The IRB determined that no consent was necessary because the prospective aspect of the study was from a registry and the rest was retrospective chart review. The Strengthening the Reporting of Observational Studies in Epidemiology (STROBE) Statement: guidelines for reporting observational studies were followed as much as possible (26).

Tumor diagnosis was based on histopathology, since the current World Health Organization guidelines were not available during most of the study (27). Analysis for isocitrate dehydrogenase (IDH) and for detection of O-6-Methylguanine-DNA Methyltransferase (MGMT) promoter methylation was unavailable at our institution before 2019. Therefore, tumor blocks and slides from 2010 to 2019 were retrospectively analyzed for these markers. This analysis was supported by a grant from a hospital research foundation (Memorial Medical Center Foundation Grant 2018-31).

No part of this study or report was produced with artificial intelligence.

### 2.2 Participants

**Figure 1:**
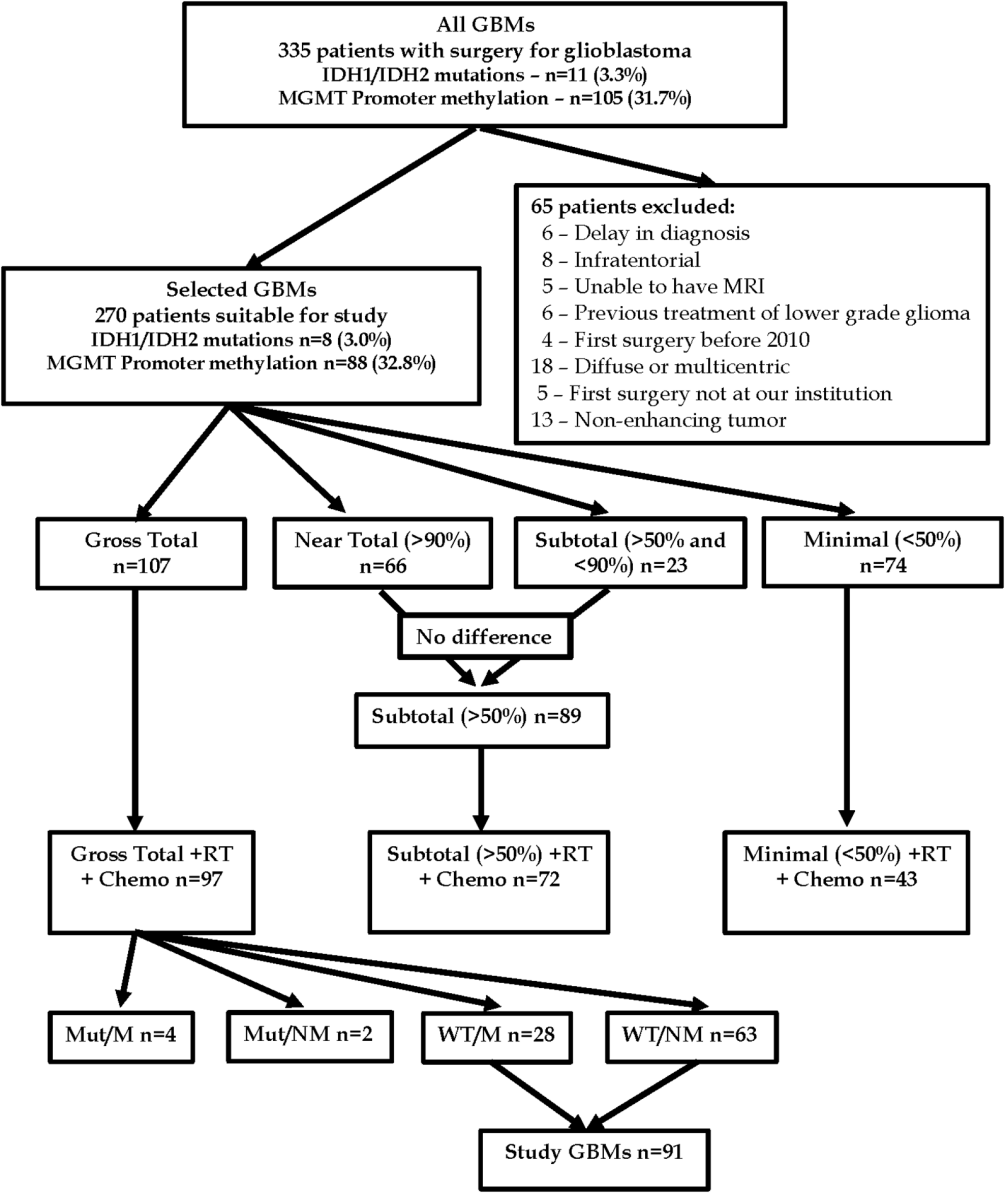
Flow diagram of patients in the current study: Mut/M = IDH-mutant and MGMT promoter methylated, Mut/NM = IDH-mutant and MGMT promoter not methylated, WT/M = IDH-wildtype and MGMT promoter methylated, WT/M = IDH-wildtype and MGMT promoter not methylated, RT, radiation therapy; Chemo, chemotherapy.

Included in this study are all individuals who have undergone surgery with histopathology consistent with glioblastoma. This group is named “All GBMs”. Because this study was intended to use overall survival outcomes and extent of resection, certain exclusions were necessary. A second group was created from the first group where individuals from the first group were excluded if their first surgery for tumor was not at our institution, first surgery prior to 2010, significant delay (>3months) in diagnosis from the time of presenting symptoms, infratentorial or spinal cord location, diffuse or non-contrast enhancing tumor, multi-centric or multifocal glioblastoma, inability of the patient to have a gadolinium-enhanced MRI scan, or glioblastoma arising from a previously diagnosed lower grade tumor. This second group is named “Selected GBMs”. Individuals in the second group were then classified by extent of resection, subsequent treatment with radiation and chemotherapy using a protocol that is a modification of that described by Stupp et al. (chemoradiation) (12, 28), and presence or absence of biological markers (IDH mutation status and MGMT promoter methylation status). From this second group, a third group was studied where all patients, had undergone a gross total resection of the tumor (resection of 100% of the contrast-enhancing portion of the tumor), IDH-wildtype, MGMT promoter methylation or not methylation, and full subsequent treatment with chemoradiation. This third group is named “Study Group”. Frailty was measured using the 5-factor modified frailty index (mFi-5) developed for the American College of Surgeons (29) and validated for studies of glioblastoma (22). Patients were identified as frail if their mFi-5 index was ≥2. The flow chart of patients is depicted in **Figure 1**.

### 2.3 Data recorded

Demographic data, tumor histology and biology, treatment data, survival and extent of resection were recorded. Age was recorded as the age of the individual at first diagnosis. Data concerning co-morbidities immediately prior to diagnosis, performance status, and frailty were recorded from chart review. Overall survival was calculated from the date of first histopathological diagnosis to the date of death. The date of death was determined from a search of medical records or public records.

Magnetic resonance images of the brain were obtained before surgery and within 48 hours after surgery. Extent of resection was determined by the percentage of the pre-operative contrast-enhancing portion of the tumor removed and recorded in four categories: Gross total (100% of the contrast-enhancing portion of the tumor removed), Near total (<100% and >90% of the contrast-enhancing portion of the tumor removed), Subtotal (<90% and >50% of the contrast-enhancing portion of the tumor removed), and Minimal (<50% of the contrast-enhancing portion of the tumor removed). Several patients in the Gross total resection group may have had what is now considered to be a “supratotal” resection (100% of the contrast-enhancing portion of the tumor as well as the surrounding non-enhancing infiltrative tumor regions) (30, 31), but this concept was not defined at the beginning of this study and therefore not recorded.

### 2.4 Statistics

Descriptive statistics were utilized to summarize patient characteristics, clinical features, and outcomes. We reported categorical variables as counts and percentages, and continuous variables using means, standard deviations, medians, and ranges, as appropriate. Continuous variables were compared between groups of interest using Student’s t-test or ANOVA for comparisons involving more than two groups. When ANOVA indicated a significant difference, *post hoc* pairwise comparisons were performed using Tukey’s test to adjust for multiple comparisons. Logistic Regression was used to examine the association of age with not receiving full chemoradiation treatment. Odds ratios and 95% confidence intervals are presented.

As an additional analysis, a Kaplan-Meier survival curve was generated to estimate overall survival probabilities. The log-rank test was used to compare survival distributions between groups and assess statistical significance.

Predictors of overall survival were assessed using univariate and multivariate Cox proportional hazards regression analyses. Hazard ratios (HR) and 95% confidence intervals (CI) are reported. All tests were two-sided, and a p-value of less than 0.05 was considered statistically significant. Statistical analyses were performed using SAS software, version 9.4 (SAS Institute Inc., Cary, NC, USA).

## 3 Results

There were 335 individuals who underwent a neurosurgical procedure (biopsy or resection) resulting in a histopathological diagnosis of glioblastoma(“All GBMs”). Of the 331 tumors tested for biological markers, 11 (3.3%) had IDH mutations, and 105 (31.7%) had MGMT promoter methylation. In the initial “All GBM’s” group, 65 patients were excluded from subsequent study using the exclusion criteria listed above. The remaining individuals comprised the “Selected GBMs” group of 270 patients (**Supplementary Table 1**).

### 3.1 Selected GBM’s – subset of 270 patients

The median age of diagnosis in this group was 66 (21–94) years. There were 8 patients with IDH mutations, 32.6% had MGMT promoter methylation, and 25.2% were considered frail. Regarding tumor resection, 35.9% had gross total resection, 24.4% had near total resection, 8.5% had subtotal resection, and 27.4% had minimal resection.

When assessing associations with age, we found a statistically significant difference based on presence of IDH mutation. Those with IDH mutations present were significantly younger than wild type (40.6 ± 16.2 vs 65.4 ± 12.2 years, respectively, p<0.0001). No significant difference in age was found based on MGMT promoter status (methylated 64.5 ± 14.5 vs non-methylated 64.8 ± 12.3 years, p=0.837). Age was significantly associated with frailty, with those in the frail group being significantly older than those not (62.6 ± 13.5 vs 70.9 ± 8.9 years, p<0.0001). A significant difference in age was also found between those who had gross total resection (61.8 ± 14.3 years) and those who had minimal resection (68.6 ± 10.8 years, adjusted p=0.0024).

Of the 270 patients, 237 had a death attributable to GBM with a median time to death of 8.8 months (0.1-103.7 months). Median follow up time for the 33 censored patients was 8.6 months (0.1-118.6 months).

**Figure 2:**
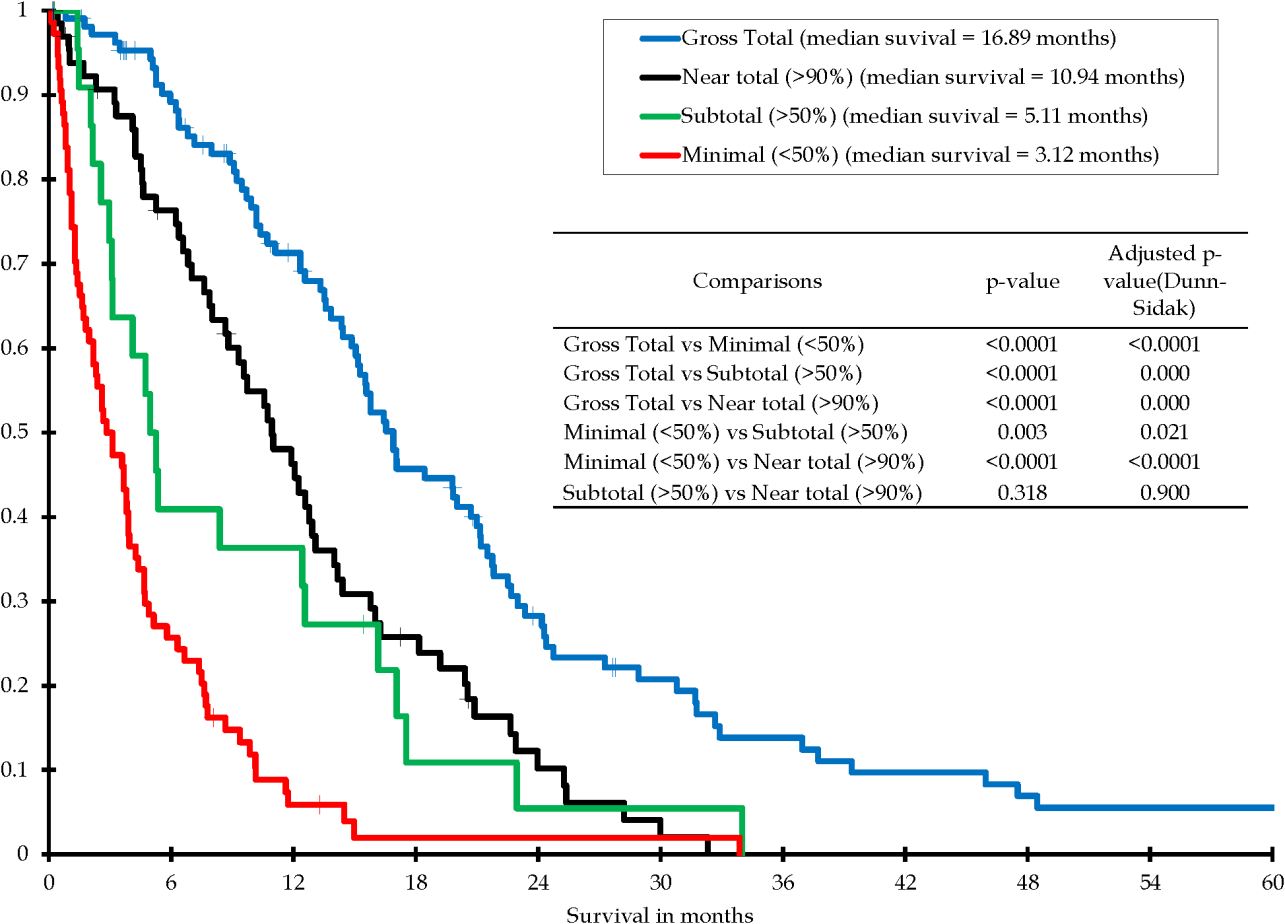
Kaplan-Meier survival distribution for all patients with histopathological diagnosis of glioblastoma, by extent of resection, with or without subsequent chemoradiation - four tiers.

Extent of resection is considered to be a major factor in survival and this was confirmed in this study. In the “Selected GBMs” group, a Kaplan-Meier survival analysis was performed on patients with a histopathological diagnosis of glioblastoma, regardless of tumor biology (IDH status or MGMT promoter status), and regardless of subsequent chemoradiation, by extent of resection using the four tiers described above. The survival curve for this group is seen in **Figure 2**. There is a significant difference between all categories of extent of resection except for the “Near total (>90% and <100% resected)” group and the “Subtotal (>50% and <90% resected)” group where there was no significant difference, with an adjusted p-value (Dunn-Sidak) of 0.900.

Of the 74 patients in the Minimal (< 50% resected) group, 55 patients had tumors that were determined to be inoperable by their neurosurgeon and therefore had stereotactic biopsy only. Of the remaining 19 patients, 6 had resections less than 50% of the tumor and the rest had stereotactic biopsies for various reasons including unsure pre-operative diagnosis, frailty, age and patient refusal to undergo a craniotomy.

In this study in most patients, post-operative radiation was prescribed following the National Comprehensive Cancer Network (NCCN) guidelines which recommend that patients 70 years-old and older receive a lesser dose of radiation (40 Gy in 15 fractions verses 60 Gy in 30 fractions for younger patients) (12). Patients who had completed a course of post-operative appropriate radiation with concomitant temozolomide chemotherapy were identified as having had complete chemoradiation.

In the “Selected GBMs” group, 58 patients out of 270 (21.5%) did not receive subsequent complete chemoradiation for unclear reasons. The reason for incomplete or no adjuvant chemoradiation was not tracked in all cases, but many patients simply refused further treatment after consulting with their neurosurgeon or neuro-oncologist. These 58 patients all had IDH-wildtype glioblastomas. Gross total resection was achieved in 10 of 58 patients (17.2%), Subtotal resection in 17 patients (29.3%), and Minimal resection in 31 patients (53.4%). Of these 58 patients, 46 received neither radiation nor chemotherapy and 12 received radiation but no chemotherapy (7 of these 12 had MGMT promoter methylated). The median mFi-5 score for the 58 patients was 1, the median pre-operative Karnofsky score was 90 and the median pre-operative ECOG score was 0. Age at diagnosis may have influenced the decision for treatment. The mean age of these 58 patients was 74.6 ± 9.9 years compared to the mean age of patients receiving full chemoradiation (62 ± 12.4 years, p<0.0001). Older age was significantly associated with the likelihood of not receiving full chemoradiation (p<0.0001). The odds ratio of not receiving full chemoradiation was 1.82 (95% CI: 1.50-2.21) for every 5-year increase in age.

### 3.2 Study GBM’s – subgroup of 91 patients

The “Selected GBMs” group was then subdivided to eliminate variability related to subsequent chemoradiation, extent of resection and tumor biology – all of which contribute to overall survival. Therefore, a new group was identified where all patients had a gross total resection, all patients had subsequent chemoradiation and all patients had tumors with IDH-wildtype. This new group of 91 patients is called “Study GBMs”. Patient demographics and clinical, tumor and treatment characteristics are summarized in **Table 1**.

**Table 1:** Study GMB patient demographics, clinical, tumor and treatment characteristics (n=91).

Age at diagnosis, years	Mean (± SD)	61.7 (12)	
		Frequency	Percent
IDH1/IDH2 mutations	Mutations present	0	0.0
	Wild-type	91	100.0
MGMT Promoter Methylation	Methylated	28	30.8
	Not methylated	63	69.2
Pre-op mFi-5	0	41	45.1
	1	36	39.6
	2	13	14.3
	3	1	1.1
	4	0	0.0
Frail	No	77	84.6
	Yes	14	15.4
Pre-op Karnofsky Score	30	0	0.0
	40	0	0.0
	50	0	0.0
	60	0	0.0
	70	1	1.1
	80	5	5.5
	90	85	93.4
	100	0	0.0
Pre-op ECOG Score	0	78	85.7
	1	10	11.0
	2	3	3.3
	3	0	0.0
	4	0	0.0
Radiation	Did not receive radiation therapy	0	0.0
	Received radiation therapy	91	100.0
Chemotherapy	Did not receive tumor chemotherapy	0	0.0
	Received tumor chemotherapy	91	100.0
Full Adjuvant Therapy	No	0	0.0
	Yes	91	100.0
Tumor Best Ever Resection	Gross Total	91	100.0
	Near total (>90%)	0	0.0
	Subtotal (>50%)	0	0.0
	Minimal (<50%)	0	0.0
Patient Status	Alive	14	15.4
	Deceased	77	84.6
Censored	Yes	18	19.8
	No	73	80.2

In this group no patients were lost to follow-up. Four individuals died from causes clearly unrelated to their brain tumor. Only one patient in the “Study GBMs” group had pre-operative Karnofsky Performance Scores <80 and every patient had pre-operative ECOG Scores ≤ 2. Statistical analysis for these scores could not be performed because of the underrepresentation of poor scores.

Due to multicollinearity of variables, unequal representation of groups, and known confounders, we analyzed this specific cohort to ensure a more homogeneous study population, allowing for a clearer evaluation of the association between age and survival. Cox proportional hazards analysis on this group was performed looking at the remaining prognostic variables of age, MGMT promoter methylation and frailty.

Of the n=91 patients, average age at diagnosis was 64 years (22–84 years), median age at diagnosis was 61.7 years, 30.8% had tumor biology where MGMT promoter was methylated, and 15.4% were considered frail. A total of n=77 patients died, with a median time to death of 18.7 months (1.7 to 66.1 months). Follow up time for the 18 censored patients ranged from 0.2 to 27.8 months, with a median follow up time of 8.1 months. Six-month survival probability estimate was 90.6% ± 3.2%.

Univariate analysis revealed age was not found to be a significant predictor of overall survival, with a hazard ratio of 1.03 (0.93-1.14) per 5-year increase in age (p=0.5380). MGMT promotor status was significantly associated with survival, with MGMT methylation experiencing a decreased hazard of death compared to non-methylated MGMT status (HR = 0.46, 95% CI: 0.26-0.82, p = 0.0082). Frailty was not found to be significant with overall survival in this cohort (Frail vs Not: HR = 1.71 [0.91-3.21], p=0.0955). After controlling for MGMT promoter methylation and frailty in multivariable analysis, age remained insignificantly associated with survival (HR=1.01 [0.99-1.03]; p=0.4009, **Table 2**).

**Table 2:** Univariate and multivariable analysis of the Study GBM group (n=91) who all had glioblastoma IDH-wildtype, gross total resection and subsequent chemoradiation.

Univariate cox proportional hazards regression				
Variable	Hazard ratio	95% Wald confidence limits		P-value
Age Unit=5	1.03	0.93	1.14	0.5380
MGMT methylated vs Not methylated	0.46	0.26	0.82	0.0083
Frail vs Not Frail	1.71	0.91	3.21	0.0955
Multivariable cox proportional hazards regression				
Variable	Hazard ratio	95% Wald confidence limits		P-value
Age Unit=5	1.01	0.99	1.03	0.4009
MGMT Methylated vs Not methylated	0.46	0.26	0.82	0.0082
Frail vs Not Frail	1.54	0.81	2.91	0.1898

**Figure 3:**
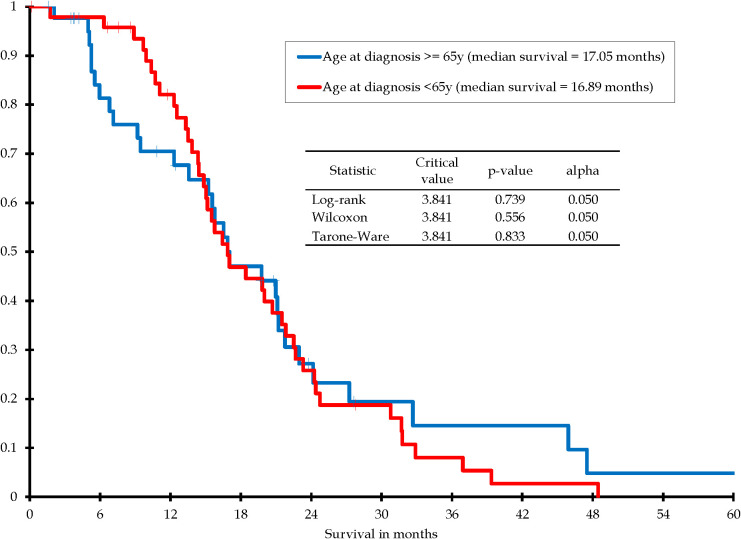
Kaplan-Meier survival distribution for patients with IDH-wildtype glioblastoma, gross total resection, subsequently treated with chemoradiation: Age at diagnosis <65 years vs. age at diagnosis ≥65 years.

Although our goal was to analyze age as a continuous variable, we felt that it might be of some interest to perform a Kaplan-Meier survival analysis at one age split. Therefore, a Kaplan-Meier analysis was performed in this group to assess survival between patients <65 years at the time of diagnosis (n=43) verses patients 65+ years at the time of diagnosis (n=48). At 6 months, the estimated survival probability was 97.9% ± 2.1% in the younger group and 81.4% ± 6.4% in those 65+ years. Median survival was 17.05 months for individuals 65+ years and 16.89 month for individuals < 65 years. No significant difference in survival distribution was found between groups (p=0.739) (**Figure 3**).

**Figure 4:**
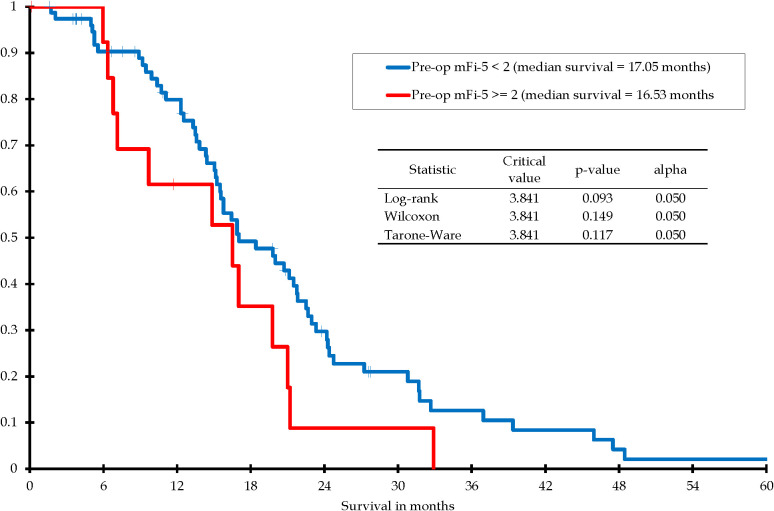
Kaplan-Meier survival distribution for patients with IDH-wildtype glioblastoma, gross total resection, subsequently treated with chemoradiation: Pre-op mFi-5 <2 vs. Pre-op mFi-5 ≥2.

The impact of frailty on survival was evaluated with Kaplan-Meier survival analysis. There were only 14 out of 91 patients (15%) who were frail in this “Study GBMs” group and there was no statistical difference in overall survival using Kaplan-Meier analysis (**Figure 4**).

## 4 Discussion

It is difficult to present arguments that contradict long-held beliefs concerning the relationship of age to length of overall survival after the initial diagnosis of glioblastoma. The notion of older age being a negative prognostic factor is well established in the medical community and it seems to be heretical to oppose this. This notion is mostly based on studies with data that that was available at the time of the study and which may or may not include other glioblastoma-related prognostic factors, some of which have been discovered only in the past few years. It is important to re-examine established beliefs and to see if they hold up in the light of new evidence.

There are several health-related factors strongly associated with older age that make an analysis of the influence of age difficult. These factors include co-morbidities, some of which are included in the mFi-5 frailty index. Factors harder to quantify are the negative accumulative health effects of lifestyle habits.

It is certainly true that in the general population, all older individuals have a higher mortality rate than younger individuals. Actuarial statistics (death from all causes) show this quite clearly with a steep increase in the death rate after age 60 years for all gender, racial, and ethnic populations (15). Glioblastoma (IDH-wildtype) is a tumor of older adults, with a median age of onset between 65–70 years (16), (66.9 years in this study), which means that more than half of the patients are in an older age group where the baseline actuarial mortality rate is significantly higher than in the younger age group. The biological mechanisms responsible for oncogenesis may be related to aging cells in the brain and aging defense systems in the body. These mechanisms may also have something to do with this tumor’s poor response to treatment. Cellular senescence is often cited as a reason for both oncogenesis and poor response to treatment. The mechanisms for poor glioblastoma treatment response in older patients have been reported in several papers (11). These studies are mostly focused on increased inflammatory response with resulting increased immunosuppression in older individuals.

There are also some age-related socio-economic factors that might affect the treatment of individuals with a glioblastoma. These factors include the willingness of individuals to pursue aggressive treatment of a tumor with a poor prognosis (23–25). In general, younger patients tend to be treated more aggressively than older patients, as was seen in our analysis. Treatment decisions concerning whether to give chemoradiation or perform complete tumor resection are heavily influenced by age. Since these treatments are also strong predictors of survival, age may appear to affect survival when it is likely a reflection of treatment selection. To address this, we focused on patients receiving standard care treatment to isolate the effect of age itself.

Previous studies that have identified younger age at diagnosis as a positive prognostic factor after the diagnosis of glioblastoma have for the most part been performed on heterogenous patient populations. These studies often included patients who have had various extent of resection ranging from biopsy to gross total resection. Numerous studies concerning the role of extent of resection on overall survival in glioblastoma, seem to indicate that it has a major influence (3, 30, 32–35). This study confirms that as well (**Figure 2**). This study was not designed to determine the factors behind the decision of the neurosurgeon to biopsy the tumor versus attempting a resection. This decision was left up to each individual neurosurgeon and the patient. In most cases the decision to attempt a resection or not was most likely based on the location of the tumor and the patient’s performance status, but it may also have had to do with the patient’s age. To nullify the differences in overall survival related to extent of resection, the “Study GBMs” group has only patients with gross total resection.

Similarly, studies that identified younger age as a positive survival factor often include patients who have not had consistent post-operative adjuvant therapy. It is well established that post-operative radiation is beneficial to survival (36–38) and it is established that post-operative chemotherapy is beneficial to survival (39). The combination of post-operative radiation and concomitant chemotherapy has been found to be even more effective (28, 39). Some current guidelines recommend withholding chemotherapy in elderly patients with MGMT promoter not methylated (12, 40). Our data shows that there is a significant number of patients in the older age groups that did not receive radiation and/or chemotherapy in the post-operative period. To nullify the differences in overall survival related to subsequent adjuvant treatment with chemoradiation, the “Study GBMs” group has only patients who had completed a course of post-operative appropriate radiation with concomitant temozolomide chemotherapy.

In the past 20 years, tumor biology has been recognized as a major factor determining response to treatment and overall survival. Starting in 2011, major registries such as the National Cancer Institute’s (NCI) Surveillance, Epidemiology, and End Results (SEER) Program began collecting data on brain molecular markers which included MGMT promoter methylation status. Then in 2018, major cancer registries began collecting data concerning IDH mutation status. Patients with histopathologically diagnosed glioblastoma and IDH mutations have been found to have a significant survival advantage over those patients with IDH-wildtype biology (27). This was also demonstrated in our study. This has been so significant that since 2021, IDH-mutant tumors are no longer considered to be glioblastomas (27). In our study, patients with histopathological features of glioblastoma and IDH-mutant biology (now classified as “astrocytoma, IDH-mutant”) were significantly younger with a median age at diagnosis of 36.48 years compared to patients with IDH-wildtype (median age at diagnosis 67.04 years). This is similar to the data recently reported by the Central Brain Tumor Registry of the United States (CBTRUS) which reported a median age of diagnosis for IDH-mutant and IDH-wildtype as 37 years and 65 years respectively (16). To nullify the differences in overall survival related to IDH status and to comply with the 2021 WHO classification, the “Study GBMs” group has only patients who had IDH-wildtype tumors.

In our 91 patient “Study GBMs” group we have therefore strived to look at a more homogeneous group where all patients were treated similarly. We found no difference in overall survival with univariate or multivariate analysis (even though some of the patients 70 years-old or older were given a lower dose of radiation than younger patients).

Finally, because of the recent interest in the concept and effect of frailty, we considered this factor as well. In our univariate analysis and multivariate analysis, frailty was not a significant predictor of overall survival. This finding differs from other studies where increased frailty was a factor resulting in poor overall survival (21, 22, 41–45). This difference may be explained by the limitations of a small sample size and that in our study, very frail patients were probably not selected to undergo an attempt at gross total resection. All the patients in our “Study GBMs” group were selected to be in that group because they had a gross total resection.

These findings are different from previous studies which have found a significant survival advantage for younger age at diagnosis, likely because most of those studies have analyzed a heterogeneous group where the patients have had various degrees of extent of resection, various subsequent adjuvant therapies (or no subsequent therapies), frail patients mixed with non-frail patients, and where the presence or absence of brain tumor markers have not necessarily been considered (1, 2, 11). It is difficult to find any studies where a relatively more homogeneous group has been studied where all patients have had similar prognostic factors of extent of resection, adjuvant therapy, brain tumor markers and frailty and the only variable was age at the time of diagnosis. This study is an attempt to look at such a group.

Other studies have suggested that frailty may be a better predictor of poor outcome than age (22, 42, 46). Although increased frequency of frailty is associated with increased age, there is not a direct correlation. There are many older individuals who are quite healthy and not frail, just as there are many young individuals who are very unhealthy and very frail. Frailty fails to achieve significance in this select group where frail patients are maybe less likely to be included. That does not mean that frailty is not important in a larger and more heterogenous group.

Our analysis focuses on assessing the effect of age as a continuous predictor on overall survival, rather than comparing survival between discrete treatment groups.

The subgroup restriction to IDH wildtype patients receiving gross total resection and full chemoradiotherapy intentionally minimizes treatment heterogeneity and associated confounding variables, thereby creating a relatively homogeneous population in terms of key clinical and biological factors. It is important to firmly establish whether advanced age by itself is a poor prognostic factor in patients with glioblastoma. The procedures and therapies for treating glioblastoma are not without risk and the costs of treatment are considerable. If the risk/benefit ratio is unfavorable in older patients then perhaps it is right to withhold care. On the other hand, if the benefit outweighs the risk and treatment is still withheld based on advanced age, then caregivers can be accused of ageism. There are several reports where authors have advocated for the aggressive treatment of glioblastoma in older patients (10, 14, 24, 47).

### 4.1 Limitations

This is a small, single-center series and as the patients were subdivided into more homogenous groups, the numbers became smaller which may be underpowered and limit the ability to detect statistically significant differences concerning the factor of age. By establishing a group for analysis with prognostic factors in common (IDH-wildtype, gross total resection, complete post-operative adjuvant therapy) our intention was to cancel out these factors and focus on age. However, by doing so we might have created a selection bias since neurosurgeons, patients and families are often unwilling to attempt extensive resection or full adjuvant chemoradiation in an elderly patient. Studying a smaller subgroup may make it harder to apply the results to all patients. However, focusing on this group helps us get clearer, more reliable results by reducing differences in treatment. Our goal is to understand how age affects survival in a specific group of patients getting standard treatment, which is important for clinical care.

While this is a relatively small, single-center study, it highlights an important clinical observation that age may not be independently associated with survival when other key factors are equalized. Larger, multi-institutional datasets are needed to confirm this finding, but sharing these early results is important. This study adds to the evidence base and may help guide more equitable treatment of older patients while prompting further investigation on a broader scale.

Replicating this study in a multicenter or international cohort would also help confirm that the findings are not unique to our single institution’s protocols or patient demographics. Larger sample sizes could also provide more statistical power when examining subgroups.

## 5 Conclusions

It has been widely observed over the past century that older individuals with glioblastoma have a poorer prognosis compared to younger individuals, but most of these observations were made of patients with glioblastoma diagnosed by histopathology only and without regard to IDH mutation status. Patients with IDH-mutant tumors are much younger and respond better than those with IDH-wildtype. If one looks at only patients with IDH-wildtype glioblastoma who have similar extent of resection and similar adjuvant therapy, the differences in survival related to age disappear. Our study population is too small to look at the influence of age on other subgroups where less than gross total resection was achieved or less than optimal adjuvant therapy. This study has implications for treatment of glioblastoma in elderly patients. Decisions regarding surgery, radiation and chemotherapy should be made based on factors specific to each patient such as comorbidities, frailty, tumor location and functional status. As a future direction of inquiry, one could explore interventions aimed at reducing frailty, which may positively influence outcomes in older patients with glioblastoma. Beyond overall survival, one should consider studying quality of life, cognitive function, and postoperative recovery trajectories. These are especially important when making treatment decisions in older populations where aggressive treatment may impact the quality of life more substantially.

Age, by itself and independent of other prognostic factors, may not be a reliable predictive factor for survival in individuals with glioblastoma. Patients should not be denied treatment based on age alone.

## Data Availability

The original contributions presented in the study are included in the article/**Supplementary Material**. Further inquiries can be directed to the corresponding author.
