# Supplementary material for: Age is not a significant predictor of survival in patients with IDH-wildtype glioblastoma that undergo gross total resection and complete adjuvant chemoradiation
Source: Front Oncol. 2025 Sep 10;15:1657867. doi: 10.3389/fonc.2025.1657867 (PMC12457138; doi:10.3389/fonc.2025.1657867)
Supplement: Supplementary file 1 [file Table1.docx]

| Supplemental Table 1. De-identified Patient Data | | | | | | | | | | | |
| --- | --- | --- | --- | --- | --- | --- | --- | --- | --- | --- | --- |
| Patient | Died | IDH1/IDH2 mutations | MGMT promoter methylation | Radiation for tumor | Chemotherapy for tumor | Age at first diagnosis (Years) | Survival in months | Best Extent of Resection | mFi-5 Pre-op | Karnofsky Score pre-op | ECOG Score Pre-op |
| 1 | TRUE | Mutations present | Not methylated | Received radiation therapy | Received tumor chemotherapy | 21.04 | 37.72 | Gross Total | 0 | 90 | 0 |
| 2 | TRUE | Wild-type | Methylated | Received radiation therapy | Received tumor chemotherapy | 22.47 | 1.74 | Gross Total | 0 | 90 | 0 |
| 3 | TRUE | Wild-type | Methylated | Received radiation therapy | Received tumor chemotherapy | 23.5 | 39.36 | Gross Total | 0 | 90 | 0 |
| 4 | TRUE | Mutations present | Methylated | Received radiation therapy | Received tumor chemotherapy | 28.8 | 6.37 | Gross Total | 0 | 90 | 0 |
| 5 | FALSE | Wild-type | Not methylated | Received radiation therapy | Received tumor chemotherapy | 28.98 | 15.44 | Subtotal (>50%) | 0 | 90 | 0 |
| 6 | TRUE | Wild-type | Not methylated | Received radiation therapy | Received tumor chemotherapy | 29.25 | 10.38 | Gross Total | 0 | 90 | 0 |
| 7 | TRUE | Mutations present | Methylated | Received radiation therapy | Received tumor chemotherapy | 31.29 | 72.9 | Gross Total | 0 | 90 | 0 |
| 8 | TRUE | Wild-type | Methylated | Received radiation therapy | Received tumor chemotherapy | 31.66 | 5.13 | Minimal (<50%) | 0 | 90 | 0 |
| 9 | TRUE | Wild-type | Not methylated | Received radiation therapy | Received tumor chemotherapy | 34.94 | 15.05 | Gross Total | 0 | 90 | 0 |
| 10 | TRUE | Mutations present | Methylated | Received radiation therapy | Received tumor chemotherapy | 35.07 | 28.91 | Gross Total | 0 | 90 | 0 |
| 11 | TRUE | Wild-type | Not methylated | Received radiation therapy | Received tumor chemotherapy | 37.14 | 18.43 | Gross Total | 0 | 90 | 0 |
| 12 | TRUE | Wild-type | Not methylated | Received radiation therapy | Received tumor chemotherapy | 37.17 | 12.55 | Subtotal (>50%) | 0 | 80 | 1 |
| 13 | FALSE | Mutations present | Methylated | Received radiation therapy | Received tumor chemotherapy | 37.88 | 118.6 | Gross Total | 0 | 90 | 0 |
| 14 | TRUE | Wild-type | Not methylated | Received radiation therapy | Received tumor chemotherapy | 37.98 | 14.39 | Subtotal (>50%) | 1 | 100 | 0 |
| 15 | TRUE | Wild-type | Not methylated | Received radiation therapy | Received tumor chemotherapy | 38.48 | 22.67 | Gross Total | 0 | 90 | 0 |
| 16 | TRUE | Wild-type | Not methylated | Received radiation therapy | Received tumor chemotherapy | 40.31 | 31.7 | Gross Total | 1 | 90 | 1 |
| 17 | TRUE | Wild-type | Not methylated | Received radiation therapy | Received tumor chemotherapy | 42.01 | 15.15 | Gross Total | 0 | 90 | 0 |
| 18 | TRUE | Wild-type | Not methylated | Received radiation therapy | Received tumor chemotherapy | 43.82 | 20.86 | Subtotal (>50%) | 0 | 80 | 1 |
| 19 | TRUE | Mutations present | Methylated | Received radiation therapy | Received tumor chemotherapy | 44.56 | 16.16 | Subtotal (>50%) | 0 | 80 | 0 |
| 20 | TRUE | Wild-type | Not methylated | Received radiation therapy | Received tumor chemotherapy | 44.65 | 7.49 | Minimal (<50%) | 0 | 90 | 0 |
| 21 | TRUE | Wild-type | Methylated | Received radiation therapy | Received tumor chemotherapy | 44.9 | 14.49 | Minimal (<50%) | 1 | 90 | 0 |
| 22 | TRUE | Wild-type | Not methylated | Received radiation therapy | Received tumor chemotherapy | 44.93 | 7.89 | Subtotal (>50%) | 0 | 90 | 0 |
| 23 | TRUE | Wild-type | Not methylated | Received radiation therapy | Received tumor chemotherapy | 45.01 | 12.75 | Subtotal (>50%) | 0 | 90 | 0 |
| 24 | TRUE | Wild-type | Not methylated | Received radiation therapy | Received tumor chemotherapy | 47.67 | 16 | Subtotal (>50%) | 0 | 90 | 0 |
| 25 | TRUE | Wild-type | Not methylated | Received radiation therapy | Received tumor chemotherapy | 49.35 | 31.77 | Gross Total | 0 | 90 | 0 |
| 26 | TRUE | Wild-type | Methylated | Received radiation therapy | Received tumor chemotherapy | 49.74 | 23.98 | Subtotal (>50%) | 0 | 90 | 0 |
| 27 | FALSE | Wild-type | Not methylated | Received radiation therapy | Received tumor chemotherapy | 49.77 | 27.76 | Gross Total | 0 | 90 | 0 |
| 28 | TRUE | Wild-type | Methylated | Received radiation therapy | Received tumor chemotherapy | 50.07 | 36.93 | Gross Total | 0 | 90 | 0 |
| 29 | TRUE | Wild-type | Methylated | Received radiation therapy | Received tumor chemotherapy | 50.7 | 28.19 | Subtotal (>50%) | 0 | 90 | 0 |
| 30 | TRUE | Wild-type | Not methylated | Received radiation therapy | Received tumor chemotherapy | 51.04 | 9.3 | Subtotal (>50%) | 1 | 90 | 0 |
| 31 | TRUE | Wild-type | Not methylated | Received radiation therapy | Received tumor chemotherapy | 51.22 | 14.95 | Minimal (<50%) | 1 | 90 | 0 |
| 32 | TRUE | Wild-type | Not methylated | Received radiation therapy | Received tumor chemotherapy | 51.93 | 21.49 | Gross Total | 0 | 90 | 0 |
| 33 | TRUE | Wild-type | Not methylated | Did not receive radiation therapy | Did not receive tumor chemotherapy | 52.13 | 2.56 | Subtotal (>50%) | 0 | 90 | 0 |
| 34 | TRUE | Wild-type | Methylated | Received radiation therapy | Received tumor chemotherapy | 52.37 | 15.77 | Gross Total | 1 | 90 | 0 |
| 35 | FALSE | Wild-type | Not methylated | Received radiation therapy | Received tumor chemotherapy | 52.48 | 17.22 | Subtotal (>50%) | 1 | 90 | 0 |
| 36 | TRUE | Wild-type | Methylated | Received radiation therapy | Received tumor chemotherapy | 52.52 | 14.36 | Gross Total | 1 | 90 | 0 |
| 37 | TRUE | Wild-type | Not methylated | Received radiation therapy | Received tumor chemotherapy | 52.73 | 9.92 | Gross Total | 0 | 90 | 0 |
| 38 | TRUE | Wild-type | Not methylated | Received radiation therapy | Received tumor chemotherapy | 52.84 | 10.15 | Minimal (<50%) | 0 | 90 | 0 |
| 39 | TRUE | Wild-type | Not methylated | Received radiation therapy | Received tumor chemotherapy | 52.95 | 4.11 | Subtotal (>50%) | 1 | 90 | 0 |
| 40 | TRUE | Wild-type | Not methylated | Did not receive radiation therapy | Did not receive tumor chemotherapy | 53 | 1.12 | Minimal (<50%) | 1 | 60 | 2 |
| 41 | TRUE | Wild-type | Methylated | Received radiation therapy | Received tumor chemotherapy | 53.08 | 17.02 | Gross Total | 2 | 90 | 0 |
| 42 | TRUE | Wild-type | Not methylated | Received radiation therapy | Received tumor chemotherapy | 53.23 | 17.05 | Subtotal (>50%) | 1 | 90 | 0 |
| 43 | TRUE | Wild-type | Not methylated | Received radiation therapy | Received tumor chemotherapy | 53.89 | 25.36 | Subtotal (>50%) | 2 | 90 | 0 |
| 44 | TRUE | Wild-type | Not methylated | Did not receive radiation therapy | Did not receive tumor chemotherapy | 54.17 | 1.41 | Minimal (<50%) | 0 | 90 | 0 |
| 45 | TRUE | Wild-type | Methylated | Received radiation therapy | Received tumor chemotherapy | 54.21 | 4.67 | Minimal (<50%) | 0 | 90 | 0 |
| 46 | TRUE | Wild-type | Methylated | Received radiation therapy | Received tumor chemotherapy | 54.47 | 13.83 | Gross Total | 1 | 90 | 0 |
| 47 | TRUE | Wild-type | Not methylated | Received radiation therapy | Received tumor chemotherapy | 54.86 | 13.5 | Gross Total | 1 | 90 | 0 |
| 48 | TRUE | Wild-type | Not methylated | Received radiation therapy | Received tumor chemotherapy | 54.98 | 19.19 | Subtotal (>50%) | 1 | 90 | 0 |
| 49 | TRUE | Wild-type | Not methylated | Received radiation therapy | Received tumor chemotherapy | 55.46 | 11.73 | Gross Total | 2 | 90 | 1 |
| 50 | TRUE | Wild-type | Methylated | Received radiation therapy | Received tumor chemotherapy | 55.5 | 25.26 | Subtotal (>50%) | 0 | 90 | 0 |
| 51 | TRUE | Wild-type | Not methylated | Received radiation therapy | Received tumor chemotherapy | 55.58 | 23.33 | Gross Total | 1 | 90 | 0 |
| 52 | TRUE | Wild-type | Not methylated | Received radiation therapy | Received tumor chemotherapy | 55.88 | 12.55 | Gross Total | 0 | 90 | 0 |
| 53 | TRUE | Wild-type | Methylated | Received radiation therapy | Did not receive tumor chemotherapy | 56.06 | 3.68 | Gross Total | 1 | 90 | 0 |
| 54 | TRUE | Wild-type | Not methylated | Received radiation therapy | Received tumor chemotherapy | 56.25 | 14 | Subtotal (>50%) | 1 | 90 | 0 |
| 55 | TRUE | Wild-type | Not methylated | Received radiation therapy | Received tumor chemotherapy | 56.3 | 5.36 | Subtotal (>50%) | 1 | 90 | 0 |
| 56 | TRUE | Wild-type | Not methylated | Received radiation therapy | Received tumor chemotherapy | 56.42 | 24.74 | Gross Total | 1 | 90 | 0 |
| 57 | TRUE | Wild-type | Not methylated | Received radiation therapy | Received tumor chemotherapy | 56.47 | 7.62 | Subtotal (>50%) | 1 | 90 | 0 |
| 58 | TRUE | Wild-type | Not methylated | Received radiation therapy | Received tumor chemotherapy | 56.96 | 14.42 | Gross Total | 0 | 90 | 0 |
| 59 | TRUE | Wild-type | Methylated | Received radiation therapy | Received tumor chemotherapy | 56.97 | 48.46 | Gross Total | 0 | 90 | 0 |
| 60 | TRUE | Wild-type | Not methylated | Received radiation therapy | Received tumor chemotherapy | 57.05 | 24.28 | Gross Total | 0 | 90 | 0 |
| 61 | TRUE | Wild-type | Not methylated | Received radiation therapy | Received tumor chemotherapy | 57.09 | 4.57 | Subtotal (>50%) | 1 | 70 | 1 |
| 62 | FALSE | Wild-type | Not methylated | Received radiation therapy | Received tumor chemotherapy | 57.1 | 6.67 | Gross Total | 0 | 90 | 0 |
| 63 | FALSE | Wild-type | Methylated | Received radiation therapy | Received tumor chemotherapy | 57.18 | 5.32 | Subtotal (>50%) | 0 | 90 | 0 |
| 64 | TRUE | Wild-type | Not methylated | Received radiation therapy | Received tumor chemotherapy | 57.6 | 20.7 | Gross Total | 0 | 90 | 0 |
| 65 | TRUE | Wild-type | Not methylated | Received radiation therapy | Received tumor chemotherapy | 58.36 | 16.89 | Gross Total | 1 | 90 | 0 |
| 66 | TRUE | Wild-type | Not methylated | Received radiation therapy | Received tumor chemotherapy | 58.59 | 24.38 | Gross Total | 1 | 90 | 0 |
| 67 | TRUE | Wild-type | Not methylated | Received radiation therapy | Received tumor chemotherapy | 59.12 | 19.81 | Gross Total | 0 | 90 | 0 |
| 68 | FALSE | Wild-type | Not methylated | Received radiation therapy | Received tumor chemotherapy | 59.22 | 7.56 | Gross Total | 0 | 90 | 0 |
| 69 | TRUE | Wild-type | Not methylated | Did not receive radiation therapy | Did not receive tumor chemotherapy | 59.31 | 3.94 | Minimal (<50%) | 1 | 70 | 3 |
| 70 | TRUE | Wild-type | Methylated | Received radiation therapy | Received tumor chemotherapy | 59.32 | 34 | Subtotal (>50%) | 3 | 90 | 0 |
| 71 | TRUE | Wild-type | Methylated | Received radiation therapy | Received tumor chemotherapy | 59.37 | 16.43 | Gross Total | 1 | 80 | 2 |
| 72 | FALSE | Wild-type | Not methylated | Received radiation therapy | Received tumor chemotherapy | 59.56 | 8.57 | Gross Total | 0 | 90 | 0 |
| 73 | TRUE | Wild-type | Methylated | Received radiation therapy | Received tumor chemotherapy | 59.59 | 14.16 | Subtotal (>50%) | 0 | 90 | 0 |
| 74 | TRUE | Wild-type | Not methylated | Received radiation therapy | Received tumor chemotherapy | 59.9 | 14.85 | Gross Total | 2 | 80 | 1 |
| 75 | TRUE | Wild-type | Not methylated | Did not receive radiation therapy | Did not receive tumor chemotherapy | 60.03 | 1.02 | Minimal (<50%) | 1 | 40 | 4 |
| 76 | TRUE | Wild-type | Methylated | Received radiation therapy | Received tumor chemotherapy | 60.18 | 15.51 | Gross Total | 0 | 90 | 0 |
| 77 | TRUE | Wild-type | Methylated | Received radiation therapy | Received tumor chemotherapy | 60.29 | 30.78 | Gross Total | 0 | 90 | 0 |
| 78 | TRUE | Wild-type | Methylated | Received radiation therapy | Received tumor chemotherapy | 60.29 | 2.3 | Minimal (<50%) | 2 | 80 | 1 |
| 79 | TRUE | Wild-type | Not methylated | Received radiation therapy | Received tumor chemotherapy | 60.29 | 10.94 | Subtotal (>50%) | 0 | 90 | 0 |
| 80 | TRUE | Wild-type | Methylated | Did not receive radiation therapy | Did not receive tumor chemotherapy | 60.5 | 2.6 | Minimal (<50%) | 1 | 90 | 0 |
| 81 | TRUE | Wild-type | Methylated | Received radiation therapy | Received tumor chemotherapy | 60.63 | 17.51 | Subtotal (>50%) | 2 | 90 | 0 |
| 82 | TRUE | Wild-type | Not methylated | Received radiation therapy | Received tumor chemotherapy | 60.67 | 21.82 | Gross Total | 0 | 90 | 0 |
| 83 | TRUE | Wild-type | Not methylated | Received radiation therapy | Received tumor chemotherapy | 60.9 | 1.35 | Minimal (<50%) | 2 | 90 | 1 |
| 84 | TRUE | Wild-type | Not methylated | Received radiation therapy | Received tumor chemotherapy | 60.98 | 32.89 | Gross Total | 2 | 90 | 0 |
| 85 | TRUE | Wild-type | Not methylated | Received radiation therapy | Received tumor chemotherapy | 61.41 | 22.51 | Gross Total | 0 | 90 | 0 |
| 86 | TRUE | Wild-type | Not methylated | Received radiation therapy | Received tumor chemotherapy | 61.52 | 5.26 | Subtotal (>50%) | 0 | 70 | 3 |
| 87 | TRUE | Wild-type | Not methylated | Received radiation therapy | Received tumor chemotherapy | 61.55 | 12.58 | Subtotal (>50%) | 0 | 90 | 0 |
| 88 | TRUE | Wild-type | Not methylated | Received radiation therapy | Received tumor chemotherapy | 61.56 | 7.62 | Minimal (<50%) | 0 | 90 | 0 |
| 89 | TRUE | Wild-type | Not methylated | Received radiation therapy | Received tumor chemotherapy | 61.61 | 12.91 | Subtotal (>50%) | 1 | 90 | 0 |
| 90 | TRUE | Wild-type | Not methylated | Received radiation therapy | Received tumor chemotherapy | 61.76 | 1.64 | Minimal (<50%) | 1 | 90 | 1 |
| 91 | TRUE | Wild-type | Not methylated | Received radiation therapy | Received tumor chemotherapy | 61.82 | 11.89 | Subtotal (>50%) | 1 | 90 | 0 |
| 92 | TRUE | Wild-type | Methylated | Received radiation therapy | Received tumor chemotherapy | 61.91 | 20.4 | Subtotal (>50%) | 2 | 90 | 0 |
| 93 | TRUE | Wild-type | Not methylated | Received radiation therapy | Received tumor chemotherapy | 62.03 | 10.12 | Minimal (<50%) | 1 | 90 | 0 |
| 94 | TRUE | Wild-type | Not methylated | Received radiation therapy | Received tumor chemotherapy | 62.26 | 12.32 | Gross Total | 0 | 90 | 0 |
| 95 | TRUE | Wild-type | Not methylated | Received radiation therapy | Received tumor chemotherapy | 62.26 | 11.73 | Minimal (<50%) | 0 | 90 | 0 |
| 96 | TRUE | Wild-type | Not methylated | Received radiation therapy | Received tumor chemotherapy | 62.38 | 6.34 | Gross Total | 2 | 90 | 0 |
| 97 | TRUE | Mutations present | Not methylated | Received radiation therapy | Received tumor chemotherapy | 62.44 | 103.69 | Gross Total | 0 | 90 | 0 |
| 98 | TRUE | Wild-type | Not methylated | Received radiation therapy | Received tumor chemotherapy | 62.5 | 20.53 | Subtotal (>50%) | 1 | 90 | 0 |
| 99 | FALSE | Wild-type | Not methylated | Received radiation therapy | Received tumor chemotherapy | 62.66 | 0.23 | Subtotal (>50%) | 1 | 90 | 0 |
| 100 | FALSE | Wild-type | Not methylated | Received radiation therapy | Received tumor chemotherapy | 62.67 | 8.71 | Subtotal (>50%) | 1 | 90 | 0 |
| 101 | TRUE | Wild-type | Methylated | Received radiation therapy | Received tumor chemotherapy | 62.77 | 22.93 | Subtotal (>50%) | 0 | 90 | 0 |
| 102 | TRUE | Wild-type | Methylated | Received radiation therapy | Received tumor chemotherapy | 62.87 | 3.12 | Minimal (<50%) | 2 | 90 | 1 |
| 103 | TRUE | Wild-type | Methylated | Received radiation therapy | Received tumor chemotherapy | 62.98 | 9.69 | Gross Total | 2 | 90 | 0 |
| 104 | TRUE | Wild-type | Methylated | Received radiation therapy | Received tumor chemotherapy | 63.01 | 13.08 | Subtotal (>50%) | 0 | 90 | 0 |
| 105 | TRUE | Wild-type | Not methylated | Received radiation therapy | Received tumor chemotherapy | 63.06 | 20.01 | Gross Total | 0 | 90 | 0 |
| 106 | TRUE | Wild-type | Methylated | Received radiation therapy | Received tumor chemotherapy | 63.13 | 15.77 | Subtotal (>50%) | 1 | 90 | 0 |
| 107 | TRUE | Wild-type | Not methylated | Received radiation therapy | Received tumor chemotherapy | 63.39 | 4.67 | Minimal (<50%) | 0 | 90 | 1 |
| 108 | TRUE | Wild-type | Not methylated | Received radiation therapy | Received tumor chemotherapy | 63.42 | 2.6 | Minimal (<50%) | 1 | 90 | 0 |
| 109 | TRUE | Wild-type | Methylated | Received radiation therapy | Received tumor chemotherapy | 63.44 | 7.36 | Minimal (<50%) | 2 | 90 | 0 |
| 110 | TRUE | Wild-type | Not methylated | Received radiation therapy | Received tumor chemotherapy | 63.46 | 10.71 | Gross Total | 0 | 90 | 0 |
| 111 | FALSE | Wild-type | Not methylated | Received radiation therapy | Received tumor chemotherapy | 63.57 | 13.27 | Minimal (<50%) | 1 | 90 | 0 |
| 112 | TRUE | Wild-type | Not methylated | Received radiation therapy | Received tumor chemotherapy | 63.91 | 13.31 | Gross Total | 1 | 90 | 0 |
| 113 | FALSE | Wild-type | Methylated | Received radiation therapy | Received tumor chemotherapy | 64.1 | 0.16 | Gross Total | 2 | 90 | 0 |
| 114 | TRUE | Wild-type | Not methylated | Received radiation therapy | Received tumor chemotherapy | 64.18 | 9.86 | Minimal (<50%) | 1 | 80 | 2 |
| 115 | TRUE | Wild-type | Not methylated | Did not receive radiation therapy | Did not receive tumor chemotherapy | 64.44 | 0.23 | Minimal (<50%) | 2 | 70 | 0 |
| 116 | TRUE | Wild-type | Not methylated | Received radiation therapy | Received tumor chemotherapy | 64.67 | 11.1 | Gross Total | 1 | 90 | 0 |
| 117 | TRUE | Wild-type | Not methylated | Received radiation therapy | Received tumor chemotherapy | 64.84 | 12.25 | Subtotal (>50%) | 1 | 90 | 1 |
| 118 | TRUE | Wild-type | Not methylated | Received radiation therapy | Received tumor chemotherapy | 64.87 | 8.9 | Gross Total | 1 | 90 | 0 |
| 119 | TRUE | Wild-type | Not methylated | Received radiation therapy | Received tumor chemotherapy | 64.95 | 29.96 | Subtotal (>50%) | 2 | 90 | 0 |
| 120 | TRUE | Wild-type | Not methylated | Received radiation therapy | Received tumor chemotherapy | 65.26 | 32.3 | Subtotal (>50%) | 0 | 90 | 0 |
| 121 | TRUE | Wild-type | Not methylated | Received radiation therapy | Received tumor chemotherapy | 65.39 | 21.19 | Gross Total | 2 | 90 | 0 |
| 122 | TRUE | Wild-type | Methylated | Received radiation therapy | Received tumor chemotherapy | 65.46 | 10.55 | Subtotal (>50%) | 0 | 90 | 1 |
| 123 | TRUE | Wild-type | Not methylated | Received radiation therapy | Received tumor chemotherapy | 65.47 | 21.13 | Gross Total | 0 | 90 | 0 |
| 124 | FALSE | Wild-type | Not methylated | Received radiation therapy | Received tumor chemotherapy | 65.49 | 2.37 | Subtotal (>50%) | 1 | 90 | 0 |
| 125 | TRUE | Wild-type | Not methylated | Received radiation therapy | Received tumor chemotherapy | 65.59 | 24.18 | Gross Total | 1 | 90 | 0 |
| 126 | TRUE | Wild-type | Not methylated | Received radiation therapy | Received tumor chemotherapy | 65.6 | 22.64 | Subtotal (>50%) | 0 | 90 | 0 |
| 127 | TRUE | Wild-type | Not methylated | Received radiation therapy | Received tumor chemotherapy | 65.76 | 7.69 | Minimal (<50%) | 2 | 90 | 0 |
| 128 | TRUE | Wild-type | Not methylated | Received radiation therapy | Received tumor chemotherapy | 65.99 | 8.38 | Subtotal (>50%) | 0 | 90 | 0 |
| 129 | TRUE | Wild-type | Not methylated | Received radiation therapy | Received tumor chemotherapy | 66.26 | 3.81 | Minimal (<50%) | 0 | 90 | 0 |
| 130 | TRUE | Wild-type | Methylated | Received radiation therapy | Received tumor chemotherapy | 66.53 | 7 | Subtotal (>50%) | 2 | 70 | 2 |
| 131 | TRUE | Wild-type | Methylated | Received radiation therapy | Received tumor chemotherapy | 66.54 | 2.1 | Gross Total | 1 | 90 | 0 |
| 132 | TRUE | Wild-type | Methylated | Received radiation therapy | Received tumor chemotherapy | 66.55 | 47.51 | Gross Total | 1 | 90 | 0 |
| 133 | TRUE | Wild-type | Not methylated | Did not receive radiation therapy | Did not receive tumor chemotherapy | 66.65 | 0.56 | Minimal (<50%) | 0 | 90 | 0 |
| 134 | TRUE | Wild-type | Not methylated | Received radiation therapy | Received tumor chemotherapy | 66.86 | 1.81 | Minimal (<50%) | 1 | 90 | 0 |
| 135 | TRUE | Wild-type | Methylated | Received radiation therapy | Received tumor chemotherapy | 66.93 | 19.78 | Gross Total | 3 | 80 | 1 |
| 136 | TRUE | Wild-type | Methylated | Received radiation therapy | Received tumor chemotherapy | 66.93 | 12.42 | Subtotal (>50%) | 1 | 90 | 0 |
| 137 | TRUE | Wild-type | Methylated | Received radiation therapy | Received tumor chemotherapy | 66.94 | 3.91 | Minimal (<50%) | 2 | 90 | 0 |
| 138 | TRUE | Wild-type | Not methylated | Did not receive radiation therapy | Did not receive tumor chemotherapy | 66.98 | 6.21 | Subtotal (>50%) | 1 | 90 | 0 |
| 139 | FALSE | Wild-type | Methylated | Received radiation therapy | Received tumor chemotherapy | 67.09 | 20.57 | Subtotal (>50%) | 1 | 80 | 1 |
| 140 | TRUE | Wild-type | Not methylated | Did not receive radiation therapy | Did not receive tumor chemotherapy | 67.12 | 4.5 | Subtotal (>50%) | 0 | 90 | 0 |
| 141 | FALSE | Wild-type | Not methylated | Received radiation therapy | Received tumor chemotherapy | 67.23 | 10.84 | Gross Total | 1 | 80 | 1 |
| 142 | TRUE | Wild-type | Not methylated | Received radiation therapy | Received tumor chemotherapy | 67.23 | 7.13 | Gross Total | 2 | 80 | 2 |
| 143 | TRUE | Wild-type | Not methylated | Did not receive radiation therapy | Did not receive tumor chemotherapy | 67.3 | 0.26 | Subtotal (>50%) | 2 | 90 | 0 |
| 144 | TRUE | Wild-type | Not methylated | Received radiation therapy | Received tumor chemotherapy | 67.33 | 4.63 | Subtotal (>50%) | 2 | 70 | 3 |
| 145 | TRUE | Wild-type | Not methylated | Did not receive radiation therapy | Did not receive tumor chemotherapy | 67.38 | 0.62 | Minimal (<50%) | 1 | 80 | 1 |
| 146 | TRUE | Wild-type | Methylated | Received radiation therapy | Received tumor chemotherapy | 67.41 | 66.14 | Gross Total | 1 | 90 | 0 |
| 147 | TRUE | Wild-type | Not methylated | Did not receive radiation therapy | Did not receive tumor chemotherapy | 67.45 | 3.22 | Subtotal (>50%) | 1 | 100 | 0 |
| 148 | TRUE | Wild-type | Not methylated | Received radiation therapy | Received tumor chemotherapy | 67.55 | 27.24 | Gross Total | 0 | 90 | 0 |
| 149 | TRUE | Wild-type | Not methylated | Received radiation therapy | Received tumor chemotherapy | 67.71 | 17.05 | Gross Total | 1 | 90 | 0 |
| 150 | TRUE | Wild-type | Not methylated | Received radiation therapy | Received tumor chemotherapy | 67.72 | 7.79 | Minimal (<50%) | 0 | 90 | 0 |
| 151 | TRUE | Wild-type | Methylated | Received radiation therapy | Received tumor chemotherapy | 67.78 | 4.27 | Minimal (<50%) | 2 | 90 | 0 |
| 152 | TRUE | Wild-type | Not methylated | Received radiation therapy | Received tumor chemotherapy | 67.79 | 5.95 | Gross Total | 2 | 90 | 0 |
| 153 | TRUE | Mutations present | Methylated | Received radiation therapy | Received tumor chemotherapy | 67.8 | 2.96 | Subtotal (>50%) | 0 | 90 | 0 |
| 154 | TRUE | Wild-type | Not methylated | Received radiation therapy | Received tumor chemotherapy | 68.01 | 5.26 | Subtotal (>50%) | 2 | 80 | 1 |
| 155 | FALSE | Wild-type | Not methylated | Received radiation therapy | Received tumor chemotherapy | 68.08 | 4.21 | Gross Total | 1 | 90 | 0 |
| 156 | FALSE | Wild-type | Methylated | Received radiation therapy | Received tumor chemotherapy | 68.51 | 23.75 | Gross Total | 1 | 90 | 0 |
| 157 | TRUE | Wild-type | Methylated | Received radiation therapy | Received tumor chemotherapy | 68.55 | 3.68 | Minimal (<50%) | 1 | 90 | 0 |
| 158 | TRUE | Wild-type | Not methylated | Received radiation therapy | Received tumor chemotherapy | 68.59 | 5.26 | Gross Total | 0 | 90 | 1 |
| 159 | TRUE | Wild-type | Methylated | Received radiation therapy | Received tumor chemotherapy | 68.73 | 45.93 | Gross Total | 0 | 90 | 0 |
| 160 | TRUE | Wild-type | Not methylated | Received radiation therapy | Received tumor chemotherapy | 68.81 | 3.52 | Gross Total | 1 | 90 | 0 |
| 161 | TRUE | Wild-type | Not methylated | Received radiation therapy | Received tumor chemotherapy | 68.82 | 22.9 | Subtotal (>50%) | 2 | 90 | 0 |
| 162 | TRUE | Wild-type | Not methylated | Received radiation therapy | Received tumor chemotherapy | 68.91 | 4.9 | Minimal (<50%) | 4 | 90 | 0 |
| 163 | TRUE | Wild-type | Not methylated | Received radiation therapy | Received tumor chemotherapy | 69.05 | 33.87 | Minimal (<50%) | 1 | 80 | 1 |
| 164 | TRUE | Wild-type | Not methylated | Received radiation therapy | Received tumor chemotherapy | 69.37 | 22.97 | Gross Total | 1 | 90 | 0 |
| 165 | TRUE | Wild-type | Not methylated | Did not receive radiation therapy | Did not receive tumor chemotherapy | 69.46 | 0.13 | Minimal (<50%) | 0 | 90 | 0 |
| 166 | TRUE | Wild-type | Not methylated | Received radiation therapy | Received tumor chemotherapy | 69.75 | 4.37 | Minimal (<50%) | 1 | 90 | 0 |
| 167 | TRUE | Wild-type | Not methylated | Received radiation therapy | Received tumor chemotherapy | 69.83 | 8.64 | Minimal (<50%) | 2 | 90 | 0 |
| 168 | TRUE | Wild-type | Not methylated | Received radiation therapy | Received tumor chemotherapy | 70.02 | 3.68 | Minimal (<50%) | 0 | 90 | 0 |
| 169 | TRUE | Wild-type | Not methylated | Received radiation therapy | Received tumor chemotherapy | 70.07 | 11.6 | Minimal (<50%) | 1 | 80 | 1 |
| 170 | TRUE | Wild-type | Methylated | Received radiation therapy | Received tumor chemotherapy | 70.27 | 5.78 | Minimal (<50%) | 3 | 50 | 4 |
| 171 | TRUE | Wild-type | Not methylated | Received radiation therapy | Received tumor chemotherapy | 70.4 | 8.64 | Subtotal (>50%) | 1 | 90 | 0 |
| 172 | FALSE | Wild-type | Methylated | Received radiation therapy | Received tumor chemotherapy | 70.47 | 20.8 | Gross Total | 0 | 90 | 1 |
| 173 | TRUE | Wild-type | Not methylated | Received radiation therapy | Received tumor chemotherapy | 70.51 | 1.61 | Gross Total | 1 | 90 | 0 |
| 174 | FALSE | Wild-type | Not methylated | Received radiation therapy | Did not receive tumor chemotherapy | 70.69 | 8.74 | Gross Total | 0 | 90 | 0 |
| 175 | FALSE | Wild-type | Not methylated | Received radiation therapy | Received tumor chemotherapy | 70.77 | 0.82 | Subtotal (>50%) | 1 | 90 | 0 |
| 176 | TRUE | Wild-type | Methylated | Received radiation therapy | Received tumor chemotherapy | 70.79 | 6.64 | Minimal (<50%) | 2 | 90 | 0 |
| 177 | TRUE | Wild-type | Not methylated | Received radiation therapy | Received tumor chemotherapy | 70.86 | 15.24 | Gross Total | 1 | 90 | 0 |
| 178 | TRUE | Wild-type | Methylated | Did not receive radiation therapy | Did not receive tumor chemotherapy | 70.92 | 1.12 | Minimal (<50%) | 2 | 90 | 0 |
| 179 | TRUE | Wild-type | Methylated | Did not receive radiation therapy | Did not receive tumor chemotherapy | 70.94 | 1.41 | Subtotal (>50%) | 1 | 90 | 1 |
| 180 | TRUE | Wild-type | Not methylated | Received radiation therapy | Received tumor chemotherapy | 71.03 | 16.89 | Gross Total | 1 | 90 | 0 |
| 181 | TRUE | Wild-type | Methylated | Received radiation therapy | Received tumor chemotherapy | 71.05 | 3.12 | Subtotal (>50%) | 0 | 90 | 1 |
| 182 | TRUE | Wild-type | Not methylated | Received radiation therapy | Received tumor chemotherapy | 71.06 | 5.09 | Gross Total | 1 | 90 | 0 |
| 183 | FALSE | Wild-type | Methylated | Received radiation therapy | Received tumor chemotherapy | 71.31 | 19.78 | Gross Total | 0 | 90 | 0 |
| 184 | FALSE | Wild-type | Not methylated | Received radiation therapy | Received tumor chemotherapy | 71.33 | 27.63 | Gross Total | 0 | 90 | 0 |
| 185 | TRUE | Wild-type | Not methylated | Received radiation therapy | Received tumor chemotherapy | 71.41 | 6.31 | Minimal (<50%) | 1 | 90 | 0 |
| 186 | TRUE | Wild-type | Methylated | Received radiation therapy | Received tumor chemotherapy | 71.51 | 15.77 | Gross Total | 0 | 90 | 0 |
| 187 | TRUE | Wild-type | Methylated | Received radiation therapy | Received tumor chemotherapy | 71.57 | 20.99 | Gross Total | 2 | 90 | 0 |
| 188 | TRUE | Wild-type | Methylated | Did not receive radiation therapy | Did not receive tumor chemotherapy | 71.61 | 1.12 | Minimal (<50%) | 2 | 80 | 1 |
| 189 | TRUE | Wild-type | Not methylated | Did not receive radiation therapy | Did not receive tumor chemotherapy | 71.71 | 1.02 | Minimal (<50%) | 2 | 90 | 0 |
| 190 | FALSE | Wild-type | Not methylated | Received radiation therapy | Received tumor chemotherapy | 72.15 | 3.75 | Gross Total | 1 | 70 | 2 |
| 191 | FALSE | Wild-type | Not methylated | Received radiation therapy | Received tumor chemotherapy | 72.22 | 8.05 | Minimal (<50%) | 2 | 90 | 0 |
| 192 | TRUE | Wild-type | Methylated | Received radiation therapy | Did not receive tumor chemotherapy | 72.57 | 4.24 | Subtotal (>50%) | 2 | 90 | 0 |
| 193 | TRUE | Wild-type | Not methylated | Received radiation therapy | Received tumor chemotherapy | 72.62 | 9.2 | Gross Total | 0 | 90 | 0 |
| 194 | TRUE | Wild-type | Not methylated | Received radiation therapy | Received tumor chemotherapy | 72.74 | 5.26 | Gross Total | 1 | 90 | 1 |
| 195 | TRUE | Wild-type | Not methylated | Received radiation therapy | Received tumor chemotherapy | 72.77 | 9.36 | Minimal (<50%) | 0 | 90 | 0 |
| 196 | TRUE | Wild-type | Methylated | Received radiation therapy | Did not receive tumor chemotherapy | 72.81 | 3.48 | Gross Total | 0 | 90 | 0 |
| 197 | TRUE | Wild-type | Not methylated | Did not receive radiation therapy | Did not receive tumor chemotherapy | 72.94 | 3.12 | Minimal (<50%) | 0 | 90 | 0 |
| 198 | TRUE | Wild-type | Methylated | Did not receive radiation therapy | Did not receive tumor chemotherapy | 73.02 | 3.29 | Subtotal (>50%) | 1 | 90 | 1 |
| 199 | TRUE | Wild-type | Methylated | Did not receive radiation therapy | Did not receive tumor chemotherapy | 73.1 | 1.71 | Minimal (<50%) | 3 | 70 | 2 |
| 200 | TRUE | Wild-type | Not methylated | Received radiation therapy | Received tumor chemotherapy | 73.12 | 2.17 | Minimal (<50%) | 2 | 80 | 1 |
| 201 | TRUE | Wild-type | Not methylated | Did not receive radiation therapy | Did not receive tumor chemotherapy | 73.18 | 2.14 | Subtotal (>50%) | 2 | 80 | 1 |
| 202 | TRUE | Wild-type | Methylated | Received radiation therapy | Received tumor chemotherapy | 73.19 | 9.46 | Gross Total | 1 | 90 | 0 |
| 203 | TRUE | Wild-type | Not methylated | Received radiation therapy | Received tumor chemotherapy | 73.49 | 0.92 | Minimal (<50%) | 2 | 80 | 1 |
| 204 | TRUE | Wild-type | Methylated | Received radiation therapy | Received tumor chemotherapy | 73.71 | 21.75 | Gross Total | 1 | 90 | 0 |
| 205 | TRUE | Wild-type | Not methylated | Received radiation therapy | Received tumor chemotherapy | 73.92 | 4.99 | Gross Total | 0 | 90 | 0 |
| 206 | TRUE | Wild-type | Methylated | Did not receive radiation therapy | Did not receive tumor chemotherapy | 74.08 | 10.18 | Gross Total | 2 | 90 | 0 |
| 207 | TRUE | Wild-type | Not methylated | Received radiation therapy | Received tumor chemotherapy | 74.13 | 1.48 | Subtotal (>50%) | 1 | 90 | 0 |
| 208 | TRUE | Wild-type | Not methylated | Did not receive radiation therapy | Did not receive tumor chemotherapy | 74.31 | 0.82 | Minimal (<50%) | 4 | 80 | 1 |
| 209 | TRUE | Wild-type | Not methylated | Received radiation therapy | Received tumor chemotherapy | 74.37 | 6.57 | Subtotal (>50%) | 0 | 90 | 0 |
| 210 | TRUE | Wild-type | Not methylated | Received radiation therapy | Received tumor chemotherapy | 74.41 | 12.32 | Gross Total | 0 | 90 | 0 |
| 211 | TRUE | Wild-type | Not methylated | Received radiation therapy | Received tumor chemotherapy | 74.51 | 2.17 | Minimal (<50%) | 3 | 80 | 1 |
| 212 | TRUE | Wild-type | Not methylated | Received radiation therapy | Received tumor chemotherapy | 74.54 | 8.02 | Subtotal (>50%) | 1 | 80 | 0 |
| 213 | TRUE | Wild-type | Not methylated | Received radiation therapy | Received tumor chemotherapy | 74.59 | 2.83 | Minimal (<50%) | 1 | 80 | 1 |
| 214 | TRUE | Wild-type | Not methylated | Did not receive radiation therapy | Did not receive tumor chemotherapy | 74.73 | 6.83 | Subtotal (>50%) | 1 | 90 | 1 |
| 215 | TRUE | Wild-type | Methylated | Did not receive radiation therapy | Did not receive tumor chemotherapy | 74.93 | 0.99 | Subtotal (>50%) | 2 | 70 | 3 |
| 216 | TRUE | Wild-type | Not methylated | Received radiation therapy | Received tumor chemotherapy | 75.47 | 16.53 | Gross Total | 2 | 90 | 1 |
| 217 | TRUE | Wild-type | Methylated | Received radiation therapy | Received tumor chemotherapy | 75.55 | 18.14 | Subtotal (>50%) | 1 | 90 | 0 |
| 218 | TRUE | Wild-type | Not methylated | Received radiation therapy | Received tumor chemotherapy | 75.58 | 6.37 | Subtotal (>50%) | 1 | 90 | 0 |
| 219 | TRUE | Wild-type | Not methylated | Did not receive radiation therapy | Did not receive tumor chemotherapy | 75.59 | 0.76 | Minimal (<50%) | 1 | 90 | 0 |
| 220 | TRUE | Wild-type | Not methylated | Received radiation therapy | Received tumor chemotherapy | 75.82 | 11.01 | Subtotal (>50%) | 1 | 90 | 0 |
| 221 | TRUE | Wild-type | Methylated | Did not receive radiation therapy | Did not receive tumor chemotherapy | 75.9 | 0.62 | Subtotal (>50%) | 1 | 80 | 1 |
| 222 | TRUE | Wild-type | Methylated | Received radiation therapy | Received tumor chemotherapy | 75.99 | 15.57 | Gross Total | 0 | 90 | 0 |
| 223 | TRUE | Wild-type | Not methylated | Did not receive radiation therapy | Did not receive tumor chemotherapy | 76.3 | 1.28 | Minimal (<50%) | 1 | 90 | 0 |
| 224 | TRUE | Wild-type | Not methylated | Received radiation therapy | Received tumor chemotherapy | 76.63 | 12.06 | Subtotal (>50%) | 1 | 90 | 0 |
| 225 | TRUE | Wild-type | Methylated | Received radiation therapy | Received tumor chemotherapy | 76.77 | 10.74 | Subtotal (>50%) | 1 | 90 | 1 |
| 226 | TRUE | Wild-type | Methylated | Received radiation therapy | Received tumor chemotherapy | 77.09 | 4.96 | Subtotal (>50%) | 1 | 90 | 0 |
| 227 | TRUE | Wild-type | Not methylated | Received radiation therapy | Received tumor chemotherapy | 77.48 | 1.28 | Minimal (<50%) | 2 | 90 | 0 |
| 228 | TRUE | Wild-type | Not methylated | Received radiation therapy | Received tumor chemotherapy | 77.67 | 5.55 | Gross Total | 1 | 90 | 0 |
| 229 | TRUE | Wild-type | Methylated | Received radiation therapy | Received tumor chemotherapy | 77.71 | 4.11 | Subtotal (>50%) | 2 | 80 | 1 |
| 230 | TRUE | Wild-type | Not methylated | Received radiation therapy | Received tumor chemotherapy | 77.73 | 4.73 | Subtotal (>50%) | 2 | 90 | 0 |
| 231 | TRUE | Wild-type | Not methylated | Did not receive radiation therapy | Did not receive tumor chemotherapy | 77.82 | 2.66 | Minimal (<50%) | 1 | 90 | 0 |
| 232 | FALSE | Wild-type | Methylated | Received radiation therapy | Received tumor chemotherapy | 77.95 | 3.78 | Gross Total | 1 | 90 | 1 |
| 233 | TRUE | Wild-type | Methylated | Did not receive radiation therapy | Did not receive tumor chemotherapy | 77.96 | 0.82 | Minimal (<50%) | 1 | 70 | 1 |
| 234 | TRUE | Wild-type | Not methylated | Received radiation therapy | Received tumor chemotherapy | 77.97 | 6.8 | Gross Total | 2 | 90 | 0 |
| 235 | TRUE | Wild-type | Not methylated | Did not receive radiation therapy | Did not receive tumor chemotherapy | 78.18 | 1.02 | Subtotal (>50%) | 3 | 80 | 1 |
| 236 | TRUE | Wild-type | Methylated | Received radiation therapy | Received tumor chemotherapy | 78.37 | 32.66 | Gross Total | 1 | 90 | 0 |
| 237 | TRUE | Wild-type | Not methylated | Received radiation therapy | Received tumor chemotherapy | 78.67 | 3.81 | Gross Total | 1 | 90 | 0 |
| 238 | TRUE | Wild-type | Not methylated | Received radiation therapy | Received tumor chemotherapy | 78.77 | 3.91 | Minimal (<50%) | 1 | 90 | 0 |
| 239 | TRUE | Wild-type | Methylated | Received radiation therapy | Received tumor chemotherapy | 78.8 | 8.8 | Subtotal (>50%) | 1 | 90 | 0 |
| 240 | FALSE | Wild-type | Not methylated | Received radiation therapy | Received tumor chemotherapy | 79.26 | 8.67 | Subtotal (>50%) | 2 | 90 | 0 |
| 241 | TRUE | Wild-type | Not methylated | Did not receive radiation therapy | Did not receive tumor chemotherapy | 80.02 | 1.51 | Minimal (<50%) | 2 | 70 | 2 |
| 242 | TRUE | Wild-type | Not methylated | Received radiation therapy | Received tumor chemotherapy | 80.18 | 2.3 | Subtotal (>50%) | 3 | 70 | 2 |
| 243 | TRUE | Wild-type | Not methylated | Did not receive radiation therapy | Did not receive tumor chemotherapy | 80.42 | 0.92 | Minimal (<50%) | 3 | 70 | 2 |
| 244 | TRUE | Wild-type | Not methylated | Received radiation therapy | Received tumor chemotherapy | 80.45 | 3.78 | Minimal (<50%) | 0 | 90 | 0 |
| 245 | TRUE | Wild-type | Not methylated | Did not receive radiation therapy | Did not receive tumor chemotherapy | 80.58 | 0.43 | Minimal (<50%) | 1 | 90 | 0 |
| 246 | TRUE | Wild-type | Methylated | Received radiation therapy | Did not receive tumor chemotherapy | 80.69 | 2.37 | Minimal (<50%) | 4 | 60 | 2 |
| 247 | TRUE | Wild-type | Not methylated | Did not receive radiation therapy | Did not receive tumor chemotherapy | 80.72 | 1.28 | Minimal (<50%) | 0 | 90 | 0 |
| 248 | TRUE | Wild-type | Not methylated | Did not receive radiation therapy | Did not receive tumor chemotherapy | 80.76 | 0.53 | Minimal (<50%) | 1 | 30 | 3 |
| 249 | TRUE | Wild-type | Not methylated | Received radiation therapy | Did not receive tumor chemotherapy | 80.93 | 6.21 | Gross Total | 2 | 90 | 0 |
| 250 | TRUE | Wild-type | Not methylated | Did not receive radiation therapy | Did not receive tumor chemotherapy | 81.52 | 1.71 | Subtotal (>50%) | 2 | 70 | 2 |
| 251 | TRUE | Wild-type | Not methylated | Received radiation therapy | Did not receive tumor chemotherapy | 81.82 | 9.07 | Gross Total | 0 | 90 | 0 |
| 252 | TRUE | Wild-type | Not methylated | Received radiation therapy | Received tumor chemotherapy | 83.09 | 9.56 | Subtotal (>50%) | 1 | 90 | 0 |
| 253 | FALSE | Wild-type | Not methylated | Received radiation therapy | Received tumor chemotherapy | 83.49 | 12.45 | Gross Total | 1 | 90 | 0 |
| 254 | TRUE | Wild-type | Not methylated | Did not receive radiation therapy | Did not receive tumor chemotherapy | 83.88 | 0.82 | Gross Total | 2 | 90 | 0 |
| 255 | TRUE | Wild-type | Methylated | Received radiation therapy | Received tumor chemotherapy | 83.9 | 2.07 | Subtotal (>50%) | 2 | 90 | 0 |
| 256 | TRUE | Wild-type | Not methylated | Received radiation therapy | Received tumor chemotherapy | 84.13 | 13.57 | Gross Total | 0 | 90 | 0 |
| 257 | TRUE | Wild-type | Methylated | Did not receive radiation therapy | Did not receive tumor chemotherapy | 84.15 | 0.69 | Minimal (<50%) | 2 | 80 | 1 |
| 258 | TRUE | Wild-type | Methylated | Received radiation therapy | Received tumor chemotherapy | 84.85 | 16.23 | Subtotal (>50%) | 1 | 90 | 0 |
| 259 | TRUE | Wild-type | Not methylated | Did not receive radiation therapy | Did not receive tumor chemotherapy | 85.49 | 0.43 | Minimal (<50%) | 1 | 50 | 3 |
| 260 | TRUE | Wild-type | Not methylated | Received radiation therapy | Did not receive tumor chemotherapy | 85.52 | 7.98 | Gross Total | 2 | 90 | 1 |
| 261 | TRUE | Wild-type | Methylated | Did not receive radiation therapy | Did not receive tumor chemotherapy | 86.03 | 3.25 | Gross Total | 2 | 90 | 0 |
| 262 | TRUE | Wild-type | Not methylated | Did not receive radiation therapy | Did not receive tumor chemotherapy | 86.87 | 3.09 | Subtotal (>50%) | 0 | 90 | 0 |
| 263 | TRUE | Wild-type | Not methylated | Received radiation therapy | Did not receive tumor chemotherapy | 87.22 | 4.7 | Minimal (<50%) | 1 | 90 | 0 |
| 264 | TRUE | Wild-type | Methylated | Received radiation therapy | Did not receive tumor chemotherapy | 87.84 | 9.72 | Subtotal (>50%) | 1 | 90 | 0 |
| 265 | TRUE | Wild-type | Not methylated | Received radiation therapy | Received tumor chemotherapy | 88.22 | 4.24 | Subtotal (>50%) | 0 | 90 | 0 |
| 266 | TRUE | Wild-type | Not methylated | Did not receive radiation therapy | Did not receive tumor chemotherapy | 88.44 | 0.46 | Minimal (<50%) | 3 | 80 | 2 |
| 267 | TRUE | Wild-type | Methylated | Did not receive radiation therapy | Did not receive tumor chemotherapy | 89.17 | 0.43 | Subtotal (>50%) | 1 | 90 | 0 |
| 268 | TRUE | Wild-type | Methylated | Received radiation therapy | Did not receive tumor chemotherapy | 90.91 | 3.58 | Minimal (<50%) | 2 | 60 | 3 |
| 269 | TRUE | Wild-type | Methylated | Received radiation therapy | Did not receive tumor chemotherapy | 92.7 | 10.18 | Gross Total | 2 | 90 | 1 |
| 270 | TRUE | Wild-type | Not methylated | Did not receive radiation therapy | Did not receive tumor chemotherapy | 94.77 | 1.97 | Minimal (<50%) | 0 | 90 | 0 |
